# Trace minerals source in calf starters interacts with birth weights to affect growth performance

**DOI:** 10.1038/s41598-022-23459-4

**Published:** 2022-11-05

**Authors:** M. A. Mousavi-Haghshenas, F. Hashemzadeh, G. R. Ghorbani, E. Ghasemi, H. Rafiee, M. H. Ghaffari

**Affiliations:** 1 Department of Animal Sciences, College of Agriculture, Isfahan University of Technology, Isfahan, 84156-83111 Iran; 2 Animal Science Research Department, Isfahan Agricultural and Natural Resources Research and Education Center, AREEO, Isfahan, 8174835117 Iran; 3 Institute of Animal Science, University of Bonn, 53115 Bonn, Germany

**Keywords:** Developmental biology, Physiology

## Abstract

The present study was conducted to investigate the effects of partial replacement of inorganic minerals (IM) with trace minerals in advance chelate components form in starter diets for calves of different birth weights on performance, health, and behavior of Holstein calves. Fifty-two calves were assigned to 1 of 4 treatments in a 2 × 2 factorial arrangement with two mineral sources (IM or advanced chelated minerals (ACMS)) and two birth weights (low or normal birth weight; LBW or NBW). Calves were weaned on d 56 and remained until d 71 of the study. Results showed that NBW calves had higher feed intake, withers and hip height, larger heart girth and lower fecal score than LBW calves throughout the study. Calves receiving ACMS tended to have higher feed intake, larger hip width, lower eye and nose scores, and lower rectal temperature throughout the study than IM calves. In addition, ACMS calves had larger abdominal girths at d 56 of the study compared to IM calves. Interactions between birth weights and mineral sources for preweaning average daily gain (ADG) and weaning and final BW showed that ACMS supplementation improved ADG and BW in LBW calves. Non-nutritive oral behavior was lower in ACMS calves compared to IM calves during all periods. Overall, ACMS feeding decreased non-nutritive oral behavior and improved calf health status during the study. In addition, feeding ACMS to dairy calves with LBW increased their ADG to a similar extent as to NBW calves, suggesting an improvement in their efficiency.

## Introduction

Dairy calves, like dairy cows, could benefit from supplementing their rations with trace minerals to meet high production demands and improve reproductive performance and the immune system^1^. In dairy cattle diets, inorganic trace minerals are the most common source of trace minerals; however, trace minerals bound to organic compounds (ligands) have been shown to be more bioavailable^2^. Various organically bound trace elements are commercially available as metal amino acids, metal proteinates and metal polysaccharides, and as metal amino acid chelates. An innovative chelate nano-compound technology combining organic acids and polymerization to deliver trace elements has recently been developed and patented^3^. With this method, molecules and atoms are connected and managed in the best way based on their affinity. Due to several factors, chelated nano-compounds can improve the delivery and bioavailability of various elements in acidic and alkaline environments^4^. The nano-compounds have a smaller diameter, allowing them to penetrate cell walls more easily. Due to their unique arrangement of atoms and molecules, chelated nano-compounds form structures that are more resistant to structural deformation in acidic and alkaline environments^3^.

The literature on the effects of dietary trace minerals or a single mineral on growth performance and health of dairy calves is inconclusive. Using organic trace minerals from amino acid complex sources, Osorio et al.^5^ reported that organic trace minerals from amino acid complex sources increased growth when supplemented at a high plane of nutrition, but not when supplemented at a low plane of nutrition. According to LaPierre et al.^6^, dairy calves fed milk replacer diets (MR) containing hydroxyl trace minerals had higher feed intake, wither height, and less diarrhea than calves fed MR containing sulfate trace minerals. Nevertheless, in their study, the amount of minerals in organic and non-organic sources was different, and organic sources contained more zinc and manganese.

Nevertheless, there is a lack of information in the literature investigating the effects of trace elements chelated with organic acid on the growth performance of dairy calves with different birth weights. Calf birth weight is a complex trait that is influenced by both environmental and genetic factors^7^. Calf birth weight is a critical factor in calf survival and morbidity at birth, as well as development and health in the future^8,9^. Compared to calves born at normal weight, calves born at high or low birth weight have a higher incidence of diarrhea at an earlier age^10^. According to McCorquodale et al.^11^, calves with higher birth weight exhibited lower susceptibility to disease in the first few years of life and were found to be more resistant to disease as they aged.

It was hypothesized that calves with low body weights would benefit more from organic mineral supplementation than calves with normal body weights during the pre- and post-weaning periods. Therefore, the objective of the current study was to investigate the effects of birth body weight (normal vs. low) and mineral source (organic vs. inorganic) in starter feed on performance and health scores of Holstein dairy calves.

## Materials and methods

### Animals, treatments, and management.

The study was conducted at Emdad Sepahan Goldasht Agriculture and Animal Husbandry Co. (Isfahan, Iran) from October 17, 2019, to January 21, 2020. Ethical approval for all procedures involving animals was obtained from the Animal Care and Use Committee of Isfahan University of Technology (IUT, Iran; IACUC #2019/B15) before the start of the study. All methods were performed following the Animal Care and Use Committee of the Iranian Council for Animal Care^12^. The study complies with ARRIVE guidelines for reporting in vivo experiments and all methods were performed in accordance with the relevant guidelines and regulations.

A total of 52 Holstein calves (32 males and 20 females) were separated from their mothers immediately after birth, weighed, and housed in individual pens (120 × 250 cm) with concrete floors bedded with straw that was replaced daily. In front of the pens there was an opening so that the calves had access to water and the feed buckets placed outside. The pens were under a 3-sided covered barn to protect the calves from direct sunlight and rainfall. All calves received 2 L of high-quality colostrum immediately after birth, measured with a colostrometer (Kruuse, Langeskov, Denmark), and another 2 L of colostrum 12 h after the first feeding. On d 2 and 3 of life, the calves received transition milk (4 L) in two equal-sized meals (at 0000 and 1200 h). Thereafter, healthy calves (without symptoms of diarrhea or systemic disease) were divided into two birth weights (low vs. normal weight) and two mineral sources (organic vs. inorganic) with 13 calves per treatment.

All calves received pasteurized whole milk (average composition: 3.2 ± 0.13% fat, 2.8 ± 0.01% crude protein (CP), 4.6 ± 0.15% lactose, and 11.5 ± 0.52% total solids) in steel buckets from d 1 (d 4 of age) until weaning on d 56. All calves received 4.5 L/d of milk from d 1 to 12 in 3 meals of equal volume (at 0900, 1700, and 0100 h) and 6 L/d of milk from d 13 to 51 in 2 meals of equal volume (at 0900 and 1700 h) and then 3 L/d from d 52 to 54 in 2 meals of equal volume (at 0900 and 1700 h) and 1.5 L/d from d 55–56 in 1 meal (at 0900 h) (total milk volume = 300 L) of the study. Calf health was monitored daily and sick calves were treated by a veterinarian as needed.

A random assignment was conducted among animals with different birth weights (low birth weight (LBW) or normal birth weight (NBW) were fed different mineral sources in ground starter diets (inorganic mineral sources (IM) or partial replacement (50:50 ratio) of advanced chelated minerals (ACMS). Mineral supplements for IM were added to the starter feed at a proportion of 0.3% of DM, while supplements for chelated minerals were added at a proportion of 0.3% of DM as a mixture (50:50 ratio) of IM (0.15% of DM) and ACMS (0.15% of DM). Treatments were (1) LBW calves fed IM (LBW-IM), (2) LBW calves fed ACMS (LBW-ACMS), (3) NBW calves fed IM (NBW-IM), and (4) NBW calves fed ACMS (NBW-ACMS). The organic minerals used in this study were a combination of organic acid chelated trace minerals (Co, Cr, Cu, Fe, Mn, Se and Zn) prepared by the self-assembly method in accordance with the advanced chelate compounds technology^3^. Based on patent US8288587B2 provided by Sodour Ahrar Shargh Company (Tehran, Iran), advanced chelate compounds are prepared under controlled conditions by polymerization of several organic acids^3^. As a result of the polymerization process, minerals are bound in a specific mixture based on their affinity for the specific organic acids that act as chelating agents^3^. To initiate a polymerization reaction, 10 g of carbonic acid was added to 50 mL of distilled water and placed in a blender at 30 °C. After 10 min, 6 g of malic acid was added to the mixture, followed by 4 min of mixing. Minerals were added to this solution as an initiator after 14 min, and the solution was maintained at a pressure of less than 2 bar between 30 and 40 °C. After mixing was complete, the temperature and pressure of the solution were lowered to 15 °C and 2.2 bar, respectively, to stop the reaction. After controlling these conditions for 5 min, the solution was kept at room temperature for one hour and then dried at 60 °C for eight hours so that the powder could be ground and sieved to ensure homogeneity^3^. As a result of the chelation process, very stable compounds are produced at a wide range of pH values^3^. The supplement contained per kilogram of supplement: Fe (mg/kg) = 4000, Co (mg/kg) = 800, Mn (mg/kg) = 18,000, Se (mg/kg) = 150, Zn (mg/kg) = 25,500, Cu (mg/kg) = 9000 and Cr (mg/kg) = 500 chelated sources and I (mg/kg) = 250 from inorganic source. Compared with the mineral concentration recommended in NASEM 2021^1^ (requirements mg/kg DM: Se = 0.30, Cu = 12, Mn = 40, and Zn = 55) for dairy calves during the weaning period, the current study targeted a slightly higher concentration of trace minerals in starter feed for calves because they may receive less starter feed in ground form during the weaning period.

The birth weights of LBW and NBW calves were 34.9 ± 2.4 and 42.7 ± 2.6 kg, respectively. Animal birth weights were classified according to the Berge et al.^35^. Table 1 shows the average of dry period length, gestation length, and parity of cows that gave birth to calves with normal or low birth weight. Calves had free access to clean water and starter feed throughout the study. The grain source of the starter feed was ground with a hammer mill with 2-mm holes in the screen. The starter feed (Table 2) was fed ad libitum to allow at least 10% ort. Nutrient composition of the feed is shown in Table 3. Refusal of starter feed was recorded and renewed each day after milk feeding at 09:30.

**Table 1:** Average of dry period length, gestation length, and parity of cows that gave birth to calves with low birth weight (LBW) and high birth weight (NBW).

	Dry period length (day)		Gestation length (day)		Parity (number)	
	LBW	NBW	LBW	NBW	LBW	NBW
Mean	69.14	75.68	273.80	278.92	1.16	1.62
Std. deviation	12.78	13.84	5.93	3.60	1.43	1.10

**Table 2:** Ingredients of ground starter feed.

Ingredients, % of DM	Treatment diet	
	IM^1^	ACMS^1^
Wheat straw	5.0	5.0
Barley grain	9.6	9.6
Corn grain	46.0	46.0
Soybean meal	25.6	25.6
Extruded soybean grain	2.8	2.8
Sugar beet pulp	6.3	6.3
Ca-salt fat^2^	0.95	0.95
Probiotics^3^	0.10	0.10
Sodium bicarbonate	0.47	0.47
Salt	0.38	0.38
Calcium carbonate	1.0	1.0
Sodium bentonite	0.60	0.60
Dicalcium phosphate	0.40	0.40
Magnesium oxide	0.30	0.30
Vitamin supplement^4^	0.20	0.20
Mineral supplement^5^	0.15	0.30
Mineral supplement^6^	0.15	–

^1^*ACMS* advanced chelated mineral source, *IM* inorganic minerals source.

^2^Pershiafat^+^, Pershiafat, Tehran, Iran. Composition: moisture, 2%; crude fat, 85% (C16:0, 35–38%; C18:0, 8–10%; C18:1, 40–42%; C18:2, 10–14%; C18:3, 1–2%).

^3^Bio-Romina, a commercial symbiotic (Zist Darman Mahan Co, Tehran, Iran) containing a combination of *Saccharomyces cerevisiae, Lactobacillus acidophilus, Lactobacillus plantarum, Lactobacillus casei, Lactobacillus rhamnosus, Bifidobacterium bifidum, Pediococcus acidilactici, Enterococcus faecium, Bacillus subtilis*, and yeast extract).

^4^Contained per kilogram of supplement: Vitamin A: 1,300,000 IU; Vitamin D: 300,000 IU; Vitamin E: 15,000 IU.

^5^Contained per kilogram of supplement: Fe (mg/kg) = 4000 Co (mg/kg) = 600 Mn (mg/kg) = 18,000 Se (mg/kg) = 150, Zn (mg/kg) = 25,000 Cu (mg/kg) = 9000 and I (mg/kg) = 250 from inorganic sources and Cr (mg/kg) = 250 from organic source.

^6^Contained per kilogram of supplement: Fe (mg/kg) = 4000 Co (mg/kg) = 800 Mn (mg/kg) = 18,000 Se (mg/kg) = 150, Zn (mg/kg) = 25,500 Cu (mg/kg) = 9000 and Cr (mg/kg) = 500 chelated sources and I (mg/kg) = 250 from inorganic source.

**Table 3:** Nutrients composition of ground starter feed.

Ingredients, % of DM	ACMS^1^	IM^1^
Dry matter	92.0	91.7
Organic matter	91.3	91.5
Crud protein	20.4	20.6
Ether extract	3.3	3.2
Neutral detergent fiber	18.4	18.7
Acid detergent fiber	8.1	8.2
Non-fiber carbohydrate^1^	49.2	49.0
Calcium	0.73	0.73
Phosphorus	0.45	0.45
Metabolizable energy, Mcal/kg of DM^2^	2.82	2.81
**Trace minerals, mg/kg of DM**		
Se	0.48	0.46
Cr	1.6	1.4
Cu	25	27
Mn	57	55
Zn	79	77
Co	1.7	1.8

^1^*IM* inorganic minerals source, *ACMS* advanced chelated mineral source.

^2^Estimated using NRC (2001) equations with the values from the analyses for starter.

### Sampling and analysis

Whole milk samples were collected weekly and analyzed for fat, CP, lactose, and total solids using an infrared spectrophotometer (Foss milk-o-scan, Foss Electric, Hillerød, Denmark). Throughout the study, starter feed intake and total DMI (milk plus starter feed) were determined daily and averaged weekly. Individual BW was recorded at the beginning of the experiment and on days 36, 56 (weaning), and 70 of the study. Average daily gain and feed efficiency (FE = kg BW gain/kg total DMI) were calculated for preweaning, postweaning, and the entire period. Throughout the study, samples of starter feed and refusals were collected every 2 weeks and stored at − 20 °C until chemical analysis. Subsamples of feed and refusals were thoroughly mixed, dried, ground to pass a 1-mm screen in a mill (Ogaw Seiki CO., Ltd, Tokyo, Japan), and analyzed (AOAC^13^) for DM (Method 925.40), ash (Method 942.05), ether extract (Method 920.39), CP (Method 2001.11), and neutral detergent fiber (NDF) (using heat stable alpha-amylase and sodium sulfite) and acid detergent fiber (ADF) according to Van Soest et al.^14^, with the Ankom Fiber Analyzer system (Ankom Technology, Macedon, NY). For mineral analysis, diet samples were ground to pass through a 0.5-mm sieve prior to analysis. The samples were then analyzed for Fe, Zn, Mn, Cu, and Co. Mineral content in the feed samples was determined using a ICP-AES after digestion in concentrated HNO_3_ according to AOAC^13^. The total concentration of Se and Cr in the feeds was also determined using an inductively coupled plasma mass spectrometry technique. Backfat thickness (BFT) was measured by ultrasound (SonoVet 600 V; BCF Technology Ltd., West Lothian, UK) once a week at the beginning of the experiment and on days 36, 56 (weaning), and 70 of the study, according to Schröder and Staufenbiel^15^ on an imaginary line between the hooks and pins at the sacral examination site.

The body measurements of each calf, including body length (distance between the points of shoulder and rump), withers height (distance from base of the front feet to the withers), body barrel (circumference of the abdomen before feeding), of heart girth (circumference of the chest), hip height (distance from base of the hind feet to hook bones), and hip width (distance between the points of the hook bones), were measured with a caliper on days 1, 36, 56, and 70 of the study using the method described by Kargar and Kanani^16^.

According to Terré et al.^17^, data on feeding behavior, including standing (no chewing activity), rumination (either lying or standing), lying (no chewing activity), eating, drinking (milk or water), and non-nutritional behavior (as the animal licked, tongue rolled, or ate wood shavings), were collected by direct observation of each calf for a 48-h period on two consecutive days before weaning (d 34 to 35 of the study) and after weaning (d 69 to 70 of the study). Behavioral data were visually monitored by two trained personnel who were unaware of the treatment. All activities were noted every 5 min, and each activity was assumed to continue throughout the 5-min interval between observations.

Health and fecal scores were recorded daily at 0730 h and were averaged by 15 d using the procedure of the Wisconsin-Madison Calf Health Scoring system^18,19^ as follows: Fecal score: 0 = normal, 1 = semi-formed, pasty, 2 = loose, but stays on top of bedding, and 3 = watery, sifts through bedding; nose score: 0 = normal, serous discharge; 1 = small amount of unilateral, cloudy discharge; 2 = bilateral, cloudy or excessive mucus, 3 = copious, bilateral mucopurulent nasal discharge; eye score: 0 = normal, 1 = mild ocular discharge, 2 = moderate bilateral ocular discharge, 3 = heavy ocular discharge; ear score: 0 = normal, 1 = ear flicking, 2 = slight unilateral ear drop, 3 = severe head tilt or bilateral ear droop. Calves with illnesses were monitored and if necessary, treated immediately by veterinarians.

Respiratory rate (RR, breaths per minute), heart rate (beats per minute), and rectal temperature were recorded weekly. Respiratory rate was determined by counting flank movements over a 3-min period. Rectal temperature was measured between 1400 and 1500 h with a standard digital thermometer (RT; PIC Vedodigit II, digital thermometer; Pic Solution Co., Como, Italy; with a measurement accuracy of 0.1 1C) inserted into the rectum for 20 s. Heart rate and RR were measured for one minute with a stethoscope^20^.

### Statistical analysis

Statistical analyses were conducted for 3 periods: pre-weaning, post-weaning, and the entire experiment using PROC MIXED (version 9.1; SAS Institute, Cary, NC) with the individual calf as the experimental unit. Starter feed intake, ADG, and feed efficiency were analyzed as repeated measures with weekly periods as the repeated variable using the following model: *Y*_*ijk*_ = *μ* + *BBW*_*i*_ + *MS*_*j*_ + *W*_*k*_ + (*BBW* × *W*)_*ik*_ + (*MS* × *W*)_*jk*_ + (*MS* × *BBW*)_*ij*_ + (*MS* × *BBW* × *W*)_*ijk*_ + *β*(*Xi − *$\overline{X}$) + *SEX*_*ijkl*_ + *ε*_*ijk*_ where Y_*ijk*_ is the dependent variable; µ is the overall mean; *BBW*_*i*_ is the effect of calves birth body weight, *MS*_*j*_ is the effect of mineral source, *W*_*k*_ is the effect of time, (*BBW* × *W*)_*ik*_ is the effect of the interaction between calves birth weight and time; (*MS* × *W*)_*jk*_ is the effect of the interaction between mineral source and time; (*BBW* × *MS*)_*ij*_ is the interaction between calves birth weight and mineral source; (*BBW* × *MS* × *W*)_*ijk*_ is the tripartite effect of calves birth weight, mineral source, and time; *β*(*Xi − *$\overline{X}$) is the covariate variable (initial BW, BFT, and structural data); *SEX*_*ijk*l_ is sex effect; and ε_*ijk*_ is the overall error. The autoregressive (order 1) covariance structure was the best fit for these data as determined by the lowest Akaike’s information criterion. The means were compared using least squares means adjusted by the Tukey procedure, and significant differences and tendencies were stated at *P* ≤ 0.05 and 0.05 < *P* ≤ 0.10, respectively.

## Results

**Figure 1:**
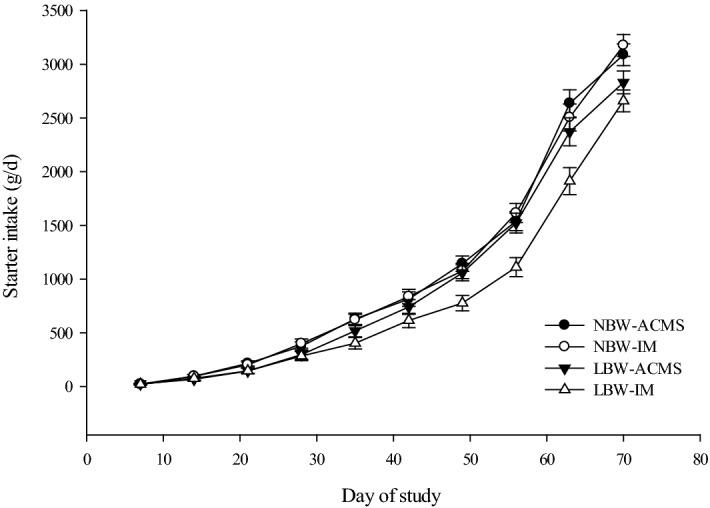
Mean starter feed intake (g/d) of calves with different birth body weights (n = 13 per treatment) fed diets with different mineral sources. Values are presented separately for normal birth weight calves fed diets containing an advanced chelated mineral source (NBW-ACMS; ●), for normal birth weight calves fed diets containing an inorganic mineral source (NBW-IM; ○), for low birth weight calves fed diets that contained an advanced chelated mineral source (LBW-ACMS; ▼), and for low birth weight calves fed diets that contained an inorganic mineral source (LBW-IM; ∆) are presented. Data are presented as mean ± SEM.

**Figure 2:**
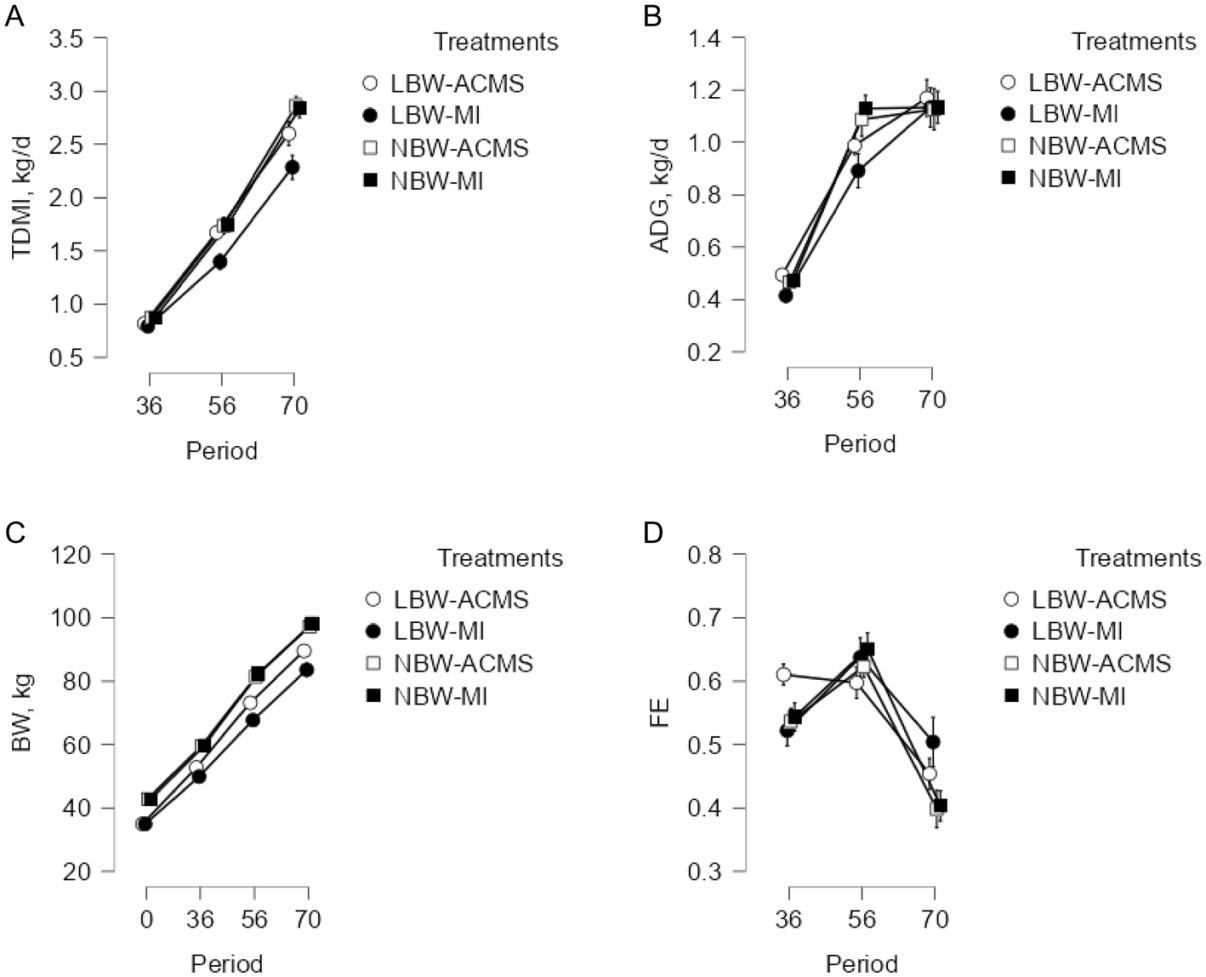
(**A**) total dry matter intake (TDMI), (**B**) average daily gain (ADG), (**C**) body weight (BW), and (**D**) feed efficiency (FE) of calves with different birth body weights (n = 13 per treatment) fed diets with different mineral sources. Values are presented separately for normal birth weight calves fed diets containing an advanced chelated mineral source (NBW-ACMS; ●), for normal birth weight calves fed diets containing an inorganic mineral source (NBW-IM; ○), for low birth weight calves fed diets that contained an advanced chelated mineral source (LBW-ACMS; ▼), and for low birth weight calves fed diets that contained an inorganic mineral source (LBW-IM; ∆) are presented. Data are presented as mean ± SEM.

Average daily starter feed intake (Fig. 1), total DMI (Fig. 2A), ADG (Fig. 2B), body weight (Fig. 2C), and feed efficiency (Fig. 2D) are shown in Table 4. Calf starter feed intakes were lower for LBW calves compared to NBW calves during the pre- and post-weaning period (P < 0.01; Table 4). For starter feed intake before weaning, birth weight and mineral source tended to interact (P = 0.09) with LBW-IM calves having lower starter feed intake than the other treatments. There was a two-way interaction between birth weight and time (P < 0.01) and mineral source and time (P = 0.02) for starter feed intake, suggesting that the effects of mineral source and birth weight on starter feed intake were even greater as calves aged. Intake of starter feed as a percent of BW was only affected by birth weight both pre- and post-weaning (P < 0.05). According to the results, NBW calves had higher starter feed intake (% of BW) before weaning but lower starter feed intake (% of BW) after weaning, resulting in no significant difference between treatments in overall starter feed intake (% of BW; Table 4). The interaction between birth weight and mineral source on total DMI was significant (P = 0.05; Table 4), with LBW-IM having the lowest total DMI among the treatments.

**Table 4:** Effects of dietary mineral source and birth body weight (BBW) on dry matter intake (DMI), average daily gain, body weight, back fat thickness, and feed efficiency of dairy calves (n = 13 per treatment).

Item	NBW^1^		LBW^1^		SEM	Treatment effects^2^						
	ACMS^1^	IM^1^	ACMS	IM		BBW	MS	BBW × MS	Time (T)	BBW × T	MS × T	BBW × MS × T
**Starter feed intake, kg/d**												
Pre-weaning	0.604	0.607	0.549	0.428	0.03	< 0.01	0.10	0.09				
Post-weaning	2.874	2.853	2.609	2.298	0.10	< 0.01	0.10	0.15				
Overall	1.056	1.054	0.959	0.800	0.04	< 0.01	0.06	0.06	< 0.01	< 0.01	0.02	0.42
**Starter feed intake, % of BW**												
Pre-weaning	1.80	2.05	1.62	1.45	0.18	0.03	0.83	0.22				
Post-weaning	2.599	2.273	2.963	2.786	0.19	0.02	0.19	0.69				
Overall	2.068	2.127	2.074	1.897	0.07	0.13	0.42	0.11	< 0.01	0.05	0.64	0.58
**Total DMI, kg/d**												
Pre-weaning	1.228	1.167	1.103	1.052	0.03	< 0.01	0.14	0.43				
Overall	1.83^a^	1.82^a^	1.70^ab^	1.50^b^	0.05	< 0.01	0.04	0.05	< 0.01	< 0.01	0.06	0.06
**Average daily gain, kg/d**												
Pre-weaning	0.77	0.80	0.74	0.65	0.03	< 0.01	0.32	0.06				
Post-weaning	1.12	1.13	1.16	1.13	0.07	0.77	0.84	0.75				
Overall	0.89	0.91	0.88	0.81	0.03	0.05	0.37	0.12	< 0.01	0.03	0.92	0.88
**Body weight, kg**												
Initial	42.8	42.7	34.9	34.9	0.71	< 0.01	0.89	0.91				
Weaning	77.4	78.5	76.9	72.0	1.82	0.21	0.19	0.04				
Final	93.3	94.5	93.3	87.8	2.05	0.29	0.20	0.04				
Overall	76.2	77.0	74.4	70.4	1.24	0.01	0.13	0.02	< 0.01	0.01	0.85	0.41
**Back fat thickness, mm**												
Pre-weaning	24.0	22.6	23.1	22.1	0.59	0.23	0.05	0.76				
Post-weaning	21.8	22.2	23.0	22.4	0.83	0.41	0.88	0.55				
Overall	23.3	22.5	23.1	22.2	0.49	0.61	0.11	0.93	< 0.01	0.34	0.05	0.24
**Feed efficiency**												
Pre-weaning	0.574	0.591	0.599	0.574	0.02	0.79	0.78	0.16				
Post-weaning	0.396	0.401	0.459	0.501	0.03	0.01	0.37	0.46				
Overall	0.512	0.529	0.552	0.547	0.01	0.04	0.66	0.61	< 0.01	0.08	0.03	0.15

^1^*IM* inorganic minerals source, *ACMS* advanced chelated mineral source, *LBW* low birth weight, *NBW* normal birth weight.

^2^Contrasts for BBW (birth body weight), MS (mineral source) and interaction (BBW × MS).

^a,b,c^Means within a row with different superscripts are significantly different (*P* < 0.05).

The interaction between birth weight and mineral source on pre-weaning ADG tended to be significant (P = 0.06; Table 4), with LBW-IM having the lowest ADG among the treatments. In addition, an interaction was found between birth weight and time on ADG (P = 0.03), suggesting that NBW calves had higher ADG compared to LBW calves before weaning, but not after weaning. There was an interaction between birth weight and mineral source for weaning and final BW (P < 0.05), because the ACMS increased BW only in LBW calves.

During the preweaning period, a tendency of interaction was observed between mineral source and time for backfat thickness (P = 0.05), indicating that ACMS calves had greater backfat thickness than IM calves. Post-weaning and total backfat thickness were not influenced by calf birth weight, mineral source, or their interaction. There was a tendency for birth weight to interact with time for feed efficiency, indicating that despite similar feed efficiency during the preweaning period, LBW calves had greater postweaning feed efficiency compared to NBW calves. In addition, we observed an interaction between mineral source and time for feed efficiency, but there was no difference between mineral source at different time points.

The interaction between birth weight and mineral source on structural growth was not significant (Table 5). Heart girth was greater (P = 0.01) in NBW calves compared to LBW calves throughout the study. In addition, there were interactions between birth weight and time for withers height and hip height (P < 0.01), suggesting that withers height and hip height were greater in NBW calves compared to LBW calves on days 36 and 70 of the study. Calves receiving ACMS tended to have greater overall hip width and heart girth (P = 0.08) compared to IM calves (P = 0.04). In addition, there was an interaction between birth weight and mineral source for calf belly girth, suggesting that ACMS calves had greater belly girth at day 56 compared to IM calves.

**Table 5:** Effects of dietary mineral source and birth body weight (BBW) on structural growth (cm) of dairy calves (n = 13 per treatment).

Item	NBW^1^		LBW^1^		SEM	Treatment effects^2^						
	ACMS	IM	ACMS^1^	IM^1^		BBW	MS	BBW × MS	Time (T)	BBW × T	MS × T	BBW × MS × T
**Body length**												
Initial	56.7	56.6	60.7	60.4	0.63	< 0.01	0.78	0.88				
d 36	64.3	64.7	64.7	64.4	0.53	0.93	0.95	0.50				
d 56	73.7	72.4	72.9	72.2	1.15	0.72	0.36	0.77				
d 70	78.2	78.5	79.0	80.6	1.11	0.30	0.39	0.55				
Overall	72.3	72.1	71.8	72.0	0.59	0.70	0.95	0.75	< 0.01	0.26	0.31	0.73
**Wither height**												
Initial	82.8	82.2	86.9	86.3	0.89	< 0.01	0.45	0.95				
d 36	88.0	87.7	90.3	88.7	0.58	0.02	0.09	0.26				
d 56	94.3	94.0	95.2	94.4	0.76	0.47	0.46	0.76				
d 70	97.0	96.5	99.4	100.1	0.54	< 0.01	0.83	0.26				
Overall	93.1	92.8	94.9	94.4	0.52	< 0.01	0.43	0.78	< 0.01	< 0.01	0.13	0.11
**Hip height**												
Initial	81.5	80.6	85.2	85.0	0.87	< 0.01	0.47	0.65				
d 36	86.6	86.5	89.0	87.4	0.58	0.02	0.15	0.17				
d 56	93.0	92.7	94.0	93.2	0.76	0.40	0.45	0.74				
d 70	95.7	95.2	98.3	98.9	0.54	< 0.01	0.99	0.31				
Overall	91.7	91.6	93.8	93.2	0.52	< 0.01	0.43	0.66	< 0.01	< 0.01	0.25	0.11
**Hip width**												
Initial	17.4	18.3	19.4	20.3	0.31	< 0.01	< 0.01	0.90				
d 36	22.3	21.9	22.2	22.1	0.23	0.70	0.35	0.43				
d 56	25.4	24.4	25.5	25.1	0.37	0.40	0.10	0.44				
d 70	28.2	27.6	28.8	28.7	0.36	0.10	0.43	0.41				
Overall	25.6	24.8	25.4	25.0	0.26	0.94	0.04	0.38	< 0.01	0.17	0.1	0.97
**Belly girth**												
Initial	80.3	80.2	83.1	85.7	1.16	< 0.01	0.27	0.25				
d 36	89.4	89.7	90.2	90.0	0.95	0.62	0.94	0.78				
d 56	108.7	103.1	104.9	100.2	2.16	0.18	0.02	0.86				
d 70	118.0	115.9	119.3	118.4	1.31	0.22	0.26	0.68				
Overall	105.3	102.8	105.0	103.4	1.16	0.91	0.08	0.70	< 0.01	0.39	0.03	0.68
**Heart girth**												
Initial	75.8	75.2	78.7	80.0	0.97	< 0.01	0.33	0.14				
d 36	83.5^b^	84.2^b^	86.9^a^	84.5^b^	0.61	0.01	0.16	0.02				
d 56	96.0	94.3	98.7	96.8	1.20	0.06	0.17	0.94				
d 70	101.9	101.3	105.5	104.0	0.90	< 0.01	0.27	0.61				
Overall	93.9	93.7	97.0	94.6	0.72	0.01	0.08	0.17	< 0.01	0.83	0.65	0.89

^1^*IM* inorganic minerals source, *ACMS* advanced chelated mineral source, *LBW* low birth weight, *NBW* normal birth weight.

^2^Contrasts for BBW (birth body weight), MS (mineral source) and interaction (BBW × MS).

^a,b,c^Means within a row with different superscripts are significantly different (*P* < 0.05).

During the preweaning and overall periods, NBW calves had lower fecal scores compared to LBW calves (P < 0.01; Table 6). Similarly, NBW calves had fewer days with a fecal score ≥ 2 compared to LBW calves. In addition, there was an interaction between birth weight and mineral source for fecal scores, suggesting that supplementation of ACMS for LBW calves resulted in a reduction in fecal scores during the preweaning period and overall, but mineral source had no effect on NBW calves.

**Table 6:** Effects of dietary mineral source and birth body weight (BBW) on health scores of dairy calves (n = 13 per treatment).

Item	NBW^1^		LBW^1^		SEM	Treatment effects^2^						
	ACMS^1^	IM^1^	ACMS	IM		BBW	MS	BBW × MS	Time (T)	BBW × T	MS × T	BBW × MS × T
**Fecal score**												
Pre-weaning	0.53^c^	0.51^c^	0.61^b^	0.69^a^	0.02	< 0.01	0.13	0.02				
Post-weaning	0.31	0.29	0.31	0.26	0.06	0.78	0.12	0.77				
Overall	0.48^c^	0.46^c^	0.54^b^	0.59^a^	0.02	< 0.01	0.50	0.04	< 0.01	0.10	< 0.01	0.27
Days with score ≥ 2	4.58	3.58	6.11	6.27	0.81	0.01	0.60	0.47				
**Nose score**												
Pre-weaning	0.33	0.40	0.27	0.37	0.03	0.08	< 0.01	0.66				
Post-weaning	0.75	0.75	0.57	0.46	0.06	< 0.01	0.32	0.36				
Overall	0.44	0.49	0.35	0.39	0.02	< 0.01	0.02	0.84	< 0.01	0.19	< 0.01	0.88
Days with score ≥ 2	8.63	10.32	5.86	7.24	1.57	0.06	0.32	0.92				
**Eye score**												
Pre-weaning	0.10	0.13	0.09	0.12	0.01	0.57	0.01	0.86				
Post-weaning	0.06	0.26	0.07	0.23	0.02	0.58	< 0.01	0.53				
Overall	0.09	0.16	0.09	0.15	0.01	0.46	< 0.01	0.65	< 0.01	0.36	< 0.01	0.03
**Ear score**												
Pre-weaning	0.05	0.04	0.07	0.10	0.01	< 0.01	0.41	0.06				
Post-weaning	0.09	0.08	0.07	0.05	0.02	0.15	0.42	0.84				
Overall	0.06	0.05	0.07	0.09	0.01	0.01	0.81	0.15	< 0.01	0.05	0.15	0.28
Days with score ≥ 2	0.98^b^	0.44^d^	0.67^c^	1.98^a^	0.35	0.08	0.28	0.01				
**Rectal temperature, °C**												
Pre-weaning	38.9	39.1	39.0	39.1	0.07	0.39	0.01	0.62				
Post-weaning	38.4	38.7	38.3	38.7	0.12	0.75	0.04	0.69				
Overall	38.81	39.05	38.89	39.04	0.07	0.62	< 0.01	0.48	< 0.01	0.22	< 0.01	0.16
**Heart rate, beat/min**												
Pre-weaning	98.6^b^	103.5^a^	96.6^c^	99.3^b^	0.43	< 0.01	< 0.01	< 0.01				
Post-weaning	84.4^c^	98.8^a^	83.2^c^	94.6^b^	0.47	< 0.01	< 0.01	< 0.01				
Overall	96.0	102.4	93.8	98.6	0.39	< 0.01	< 0.01	0.04	< 0.01	0.01	< 0.01	0.02
**Respiration rate, breath/min**												
Pre-weaning	38.2^b^	41.0^a^	41.2^a^	40.6^a^	0.52	0.01	0.05	< 0.01				
Post-weaning	31.8^b^	37.0^a^	35.2^a^	37.0^a^	0.69	< 0.01	< 0.01	0.01				
Overall	36.7^b^	40.5^a^	40.2^a^	39.7^a^	0.55	0.01	< 0.01	< 0.01	0.01	0.23	0.01	0.07

^1^*IM* inorganic minerals source, *ACMS* advanced chelated mineral source, *LBW* low birth weight, *NBW* normal birth weight.

^2^Contrasts for BBW (birth body weight), MS (mineral source) and interaction (BBW × MS).

^a,b,c^Means within a row with different superscripts are significantly different (*P* < 0.05).

During the preweaning and overall periods, NBW calves had lower fecal scores (P < 0.01) compared to LBW calves. Similarly, NBW calves had fewer days with a fecal score ≥ 2 compared to LBW calves. In addition, there was an interaction between birth weight and mineral source for fecal scores, suggesting that supplementing of ACMS to starter feed for LBW calves decreased fecal scores during the preweaning and overall periods, but mineral source had no effect on NBW calves.

The NBW calves had more nasal scores and days with nasal scores ≥ 2 throughout the study compared with LBW calves. There was a 2-way interaction between mineral source and time for nasal scores, suggesting that supplementation with ACMS decreased calf nasal scores only during the preweaning period. In addition, ACMS feeding decreased calf eye scores compared with IM. The interaction between birth weight and ear score timing (P = 0.05) suggests that NBW calves had lower ear scores during the preweaning period. In addition, an interaction between birth weight and mineral source was observed for ear drop scores prior to weaning, with ACMS fed calves having lower ear drop scores only in LBW calves. Calf birth weight had no effect on calf rectal temperature, but ACMS supplementation lowered calf rectal temperature compared with IM (P < 0.05).

There was a tendency for an interaction between birth weight and time spent eating (P = 0.08; Table 7), as NBW calves tended to spend less time eating compared with LBW calves. Calves fed ACMS spent more time eating before weaning (P = 0.07) than calves fed IM. NBW calves spent more time drinking than LBW calves throughout the study (P < 0.01). A two-way interaction between birth weight and mineral source (P = 0.07) for drinking time showed that supplementation with ACMS increased drinking time in NBW calves compared with supplementation with IM sources. Although overall standing time was longer, post-weaning lying time was lower in NBW calves compared with LBW calves. In addition, an interaction between mineral source and time was observed for lying time, which resulted in ACMS calves having less time to lying time after weaning than calves supplemented with IM sources. In addition, non-nutritive oral behavior was significantly lower in ACMS calves compared to IM calves during all time periods.

**Table 7:** Effects of dietary mineral source and birth body weight (BBW) on total time devoted to performing different behaviors (min) during 48 h of observation of dairy calves (n = 13 per treatment).

Items	NBW^1^		LBW^1^		SEM	Treatment effects^2^						
	ACMS^1^	IM^1^	ACMS	IM		BBW	MS	BBW × MS	Time (T)	BBW × T	MS × T	BBW × MS × T
**Eating (min)**												
Pre-weaning	222.1	189.9	219.1	209.5	11.45	0.51	0.07	0.33				
Post-weaning	291.7	269.4	306.4	297.5	12.51	0.09	0.22	0.59				
Overall	257.9	229.6	262.5	254.5	11.55	0.21	0.12	0.44	< 0.01	0.08	0.44	0.55
**Drinking (min)**												
Pre-weaning	50.1^a^	41.7^b^	32.6^c^	37.1^bc^	3.12	< 0.01	0.52	0.04				
Post-weaning	87.0	65.5	65.1	56.3	4.44	< 0.01	< 0.01	0.16				
Overall	68.6	53.6	48.9	46.7	3.45	< 0.01	0.02	0.07	< 0.01	0.17	< 0.01	0.98
**Ruminating (min)**												
Pre-weaning	433.7	418.4	399.1	409.5	21.74	0.31	0.90	0.55				
Post-weaning	625.2	547.8	560.9	574.0	27.91	0.49	0.25	0.11				
Overall	528.9	482.6	479.5	491.3	23.33	0.38	0.46	0.22	< 0.01	0.88	0.10	0.17
**Standing (min)**												
Pre-weaning	361.2	398.2	352.0	345.5	14.67	0.04	0.30	0.14				
Post-weaning	325.8	313.9	248.4	218.6	16.48	< 0.01	0.21	0.59				
Overall	343.3	355.8	300.0	281.7	12.84	< 0.01	0.82	0.24	< 0.01	< 0.01	0.05	0.50
**Lying (min)**												
Pre-weaning	1440.6	1445.6	1470.0	1450.6	24.74	0.49	0.77	0.62				
Post-weaning	1337.5	1441.7	1424.3	1450.9	24.76	0.05	0.01	0.12				
Overall	1389.1	1443.7	1447.2	1450.8	20.40	0.12	0.16	0.22	0.01	0.28	0.01	0.35
**Non-nutritive oral behavior (min)**												
Pre-weaning	374.9	389.1	348.7	430.7	23.18	0.73	0.04	0.14				
Post-weaning	215.6	244.8	214.8	285.6	22.53	0.37	0.03	0.35				
Overall	295.3	316.8	281.8	358.1	19.57	0.48	0.02	0.16	< 0.01	0.59	0.94	0.58

^1^*IM* inorganic minerals source, *ACMS* advanced chelated mineral source, *LBW* low birth weight, *NBW* normal birth weight.

^2^Contrasts for BBW (birth body weight), MS (mineral source) and interaction (BBW × MS).

^a,b,c^ Means within a row with different superscripts are significantly different (*P* < 0.05).

## Discussion

The current study investigated the effects of feeding starter diets with different mineral sources to calves with different birth weight on intake, growth performance, health scores, and behavior. It was hypothesized that calves with low birth weights would benefit more from organic mineral supplementation during the pre- and post-weaning periods than calves with normal birth weights. The interaction between birth weight and mineral source was significant, with LBW-IM calves having the lowest ADG and BW. The lower ADG and BW in LBW-IM calves was due to the lower intake of starter feed in LBW calves and the trend toward lower intake of starter feed in IM calves throughout the period. This result suggests that feeding ACMS to dairy calves at LBW may increase their ADG to a similar extent as in NBW calves, suggesting an improvement in their efficiency.

The greater pre-weaning ADG in LBW-ACMS calves compared to calves fed IM starter suggests that enhancing mineral bioavailability may optimize calf efficiency in early life when calves have lower immune performance^21^. Because peroxide radicals formed during periods of stress can affect glucose metabolism^22^ and minerals affect the elimination and formation of peroxide, improved bioavailability of OM increased ADG and FE during the pre-weaning period of life^5,23^. Boma and Bilkei^24^ in swine and Osorio et al.^5^ in dairy calves reported that OM improved animal performance when exposed to stressors such as adverse temperatures and transportation. Taken together, these results suggest that mineral source affects calf performance in the early days of life or under stressful conditions. To our knowledge, this is the first experiment to examine the effects of mineral source and birth weight on calf behavior. The interaction between birth weight and mineral source affected drinking time, with NBW-ACMS calves having the longest drinking time before weaning and throughout the period.

Starter feed intake, total DMI, and BW were higher in NBW calves than in LBW calves. In addition, NBW calves tended to have greater overall ADG compared to LBW calves. Higher birth weight could affect DMI as it is related to BW due to maintenance requirements^22^. Since milk intake was constant between treatments, greater starter feed intake provides more nutrients for growth in NBW calves compared to LBW calves. Yaylak et al.^25^ reported in Holstein calves that a one kg increase in birth weight resulted in a 0.93 kg increase in weaning weight. MacGregor and Casey^26^, indicated that a one kg increase in birth weight resulted in a 0.005 ± 0.0002 kg increase in pre-weaning ADG and a 0.05 ± 0.02 day decrease in weaning age because higher birth BW was associated with later calving. In contrast to our results, Berge et al.^27^ reported that ADG was higher in LBW calves during the first 28 days of life. In this study, starter feed intake was not reported between different birth weights, and the increase in ADG was attributed to calves receiving more milk per kg of BW than heavier calves. However, in our study, heavier calves received more starter feed, which resulted in more energy from the starter feed. Pabst et al.^28^ reported that birth weight had a positive relationship with performance. In other words, calves with higher birth weight had a higher growth rate than calves with lower birth weight^29–31^. In addition, calves with higher birth weight also had higher persistency than calves with lower birth weight^32,33^. Nonetheless, feed efficiency was increased in the LBW calves, indicating an improvement in energy and protein utilization efficiency, probably due to lower maintenance requirements. Consistent with our results, Bailey and Mears^34^, reported that birth weight was positively correlated with ADG and negatively with efficiency of weight gain. In contrast to our results, Garcia et al.^21^ reported that birth weight had no effect on starter feed intake and ADG of dairy calves, but NBW calves had a higher FE than LBW calves.

Overall, heart girth and hip and withers height were greater in NBW calves, which may be attributed to the greater initial value of body structure and ADG. Bailey and Mears^34^, concluded that absolute daily gain was proportional to the size of the growing mass. Thus, for cattle with the same genetic potential for gains, low birth weights will necessarily have lower growth rates than high birth weight animals and will be lighter at a given age. Overall, the results suggest that birth weight affects calf performance and LBW calves need more attention to compensate for their LBW and achieve the ideal ADG and body structure as NBW calves.

Fecal score was higher in LBW calves than in NBW calves, most likely due to the fact that LBW calves received more milk in relation to BW than NBW calves, since all calves received the same amount of milk regardless of birth weight, resulting in a higher fecal score than the heavier calves. Consistent with our results, LBW calves had more diarrhea days and a higher risk of being treated with antimicrobials than heavier calves^35^. The increase in heart rate in heavier calves could be due to higher ADG and initial feed intake. In other work with steers^36^, heart rate increased with increasing feed intake. Respiratory rate is a vital sign that can provide valuable information about disease, stress, pain, and overall health and well-being^37^. The increase in RR in LBW calves could indicate that they had more stress and lower well-being. The energy saved by the lower respiration rate could have been used to increase ADG. In agreement with our results, Burrow et al.^38^ indicated that cattle with a high ADG have a calm temperament, suggesting that heavier animals may be better able to cope with stress than lighter animals.

Contrary to our expectations, birth weight had no effect on feeding behavior; otherwise, eating time after weaning tended to be lower in NBW calves. The starter feed intake was greater in NBW calves compared to LBW calves, which increased eating and rumination time, as expected, but NBW calves increased eating and rumination rates rather than time. Consistent with our results, eating and rumination times were similar in calves with different DMI^39^. It appears that the effect of feed intake on feeding behavior in dairy calves differs from that in dairy cows and that calves with different DMI partially compensate for eating time by changing eating rate, and this issue requires further research. Drinking time increased in NBW calves in parallel with intake of starter feed. The differences in standing behavior between treatments could be explained by the fact that NBW calves spend more time drinking, which occurs while standing.

According to previous studies, starter feed intake did not differ between calves fed organic or inorganic minerals by Osorio et al.^5^, Gelsinger et al.^40^, Ma et al.^41^, and Chang et al.^42^; however, Abdollahi et al.^43^ reported that calves fed organic zinc had greater feed intake than calves fed inorganic zinc. The positive effect on DMI due to OM supplementation may be related to the higher digestibility reported in calves^43^ and dairy cows^44^ fed OM compared to IM. The higher bioavailability of OM and faster utilization and fermentation by rumen microorganisms could improve digestibility^45^. Diets high in starch could reduce cellulose digestion because of trace mineral deficiencies^46^. In this situation, the starch-degrading bacteria (fast-growing bacteria found in calves fed high-grain starter feed) consume the trace elements and increase the microbial demand for trace elements, so there are probably not enough trace elements for the cellulose-degrading bacteria (slow-growing bacteria)^46^. In the study, OM was reported to be more efficiently utilized by amylolytic bacteria and organic matter was more rapidly fermented in the rumen, resulting in increased digestibility and DMI. More recently, Chen et al.^47^ reported more rumen bacteria and higher DMI and lower rumen ammonia content in dry cows supplemented with more Zn-Met. On the other hand, inorganic forms of minerals are often offered as sulfates and oxalates and are less palatable^45^, possibly leading to lower intake.

In contrast to starter feed intake, mineral source had no effect on overall ADG and BW. Consistent with our results, Osorio et al.^5^, Gelsinger et al.^40^, Pino et al.^22^, and Abdollahi et al.^43^ reported that trace elements mineral source had no effect on BW and ADG in dairy calves during weaning and after weaning. In addition, weaning weights of calves fed zinc and manganese methionine were higher than those in the oxide treatment^48^. The mineral source had no effect on overall structural growth, except for belly girth at day 56 and overall period, which was greater in ACMS than in IM calves. The increase in belly girth could be due to the higher intake of starter feeds in ACMS calves. In general, the bioavailability of organic forms of the mineral is considered greater than that of inorganic forms^49^; therefore, we hypothesize that supplementation of OM could increase ADG and overall body structure. Replacing inorganic with organic mineral source did not alter body composition in a long-term study^22^, but OM increased withers and hip height in dairy calves at weaning (7 weeks)^5^.

Consistent with other studies^40,43,48^, feeding OM improved health status of dairy calves by lowering nasal score, eye score, rectal temperature, and heart and respiratory rates. Johnson et al.^50^ found that calves fed zinc methionine required 5.8% less medical treatment and morbidity rates decreased. Osorio et al.^5^ indicated that health scores did not differ between mineral sources (MS). However, feeding organic minerals to pregnant cows^40,47^ or calves^42,48^ improved the immune efficiency of dams and their calves. The potential health-promoting effects of OM may be related to the higher bioavailability of OM and its positive effects on the immune response via antioxidant pathways and maintaining the structural integrity of the epithelium against infections^51,52^. For example, Brugger and Windisch^53^ reported that Zn positively affects the modulation of inflammatory responses and the development of the gut microbiota, and inflammation and infection increased in zinc-deficient animals^54^. More recently, Ma et al.^41^ reported that Zn-Met supplementation improves intestinal mucosal barrier integrity.

Despite similar feed intake in the preweaning period, calves fed IM tended to have higher eating time in the pre-weaning period, indicating a lower eating rate. Lower digestibility when fed IM^43,44^ likely increases total track retention time, which in turn decreases eating rate. In the post-weaning and overall periods, calves fed IM tended to reduce starter feed intake, and this, along with a reduction in feeding rate, resulted in similar feeding times between IM- and ACMS-fed calves. Drinking time was higher in ACMS-fed calves during the post-weaning period and overall, which may be attributed to greater starter feed intake. There is a positive relationship between DMI and water intake^55^. The lying time of calves fed ACMS in the post-weaning period is likely due to the longer drinking time and the greater number of eating and rumination periods. In addition, IM calves exhibited greater non-nutritive oral behavior during all periods. Non-nutritive oral behavior is often considered an index of poor welfare because it is thought to be related to frustrated feeding activity^56^.

## Conclusion

According to our results, LBW calves had lower starter feed intake, ADG, and BW than NBW calves, resulting in LBW calves having to increase their growth rate to compensate for their low birth weight. The ACMS feeding improved starter feed intake, some skeletal growth parameters, and health status throughout the study compared to the IM feeding. Interactions between birth weight and mineral source were observed for BW and ADG, and LBW-IM had the lowest ADG and BW. The results of the study demonstrate that ACMS can be used as a useful component of calf starter diets to enhance the performance and health of dairy calves born at low birth weights. Further research is needed to determine the long-term effects of ACMS on Holstein heifer performance during weaning and first lactation.

## Data Availability

All data generated or analyzed during this study are included in this published article [and its supplementary information files].
